# Varying Patterns on Varying Scales: A Metacommunity Analysis of Nematodes in European Lakes

**DOI:** 10.1371/journal.pone.0151866

**Published:** 2016-03-23

**Authors:** Birgit Dümmer, Kai Ristau, Walter Traunspurger

**Affiliations:** Animal Ecology, Bielefeld University, Bielefeld, North Rhine-Westfalia, Germany; University of Milan-Bicocca, ITALY

## Abstract

Ecological community patterns are often extremely complex and the factors with the greatest influence on community structure have yet to be identified. In this study we used the elements of metacommunity structure (EMS) framework to characterize the metacommunities of freshwater nematodes in 16 European lakes at four geographical scales (radius ranging from 80 m to 360 km). The site characteristics associated with site scores indicative of the structuring gradient were identified using Spearman rank correlations. The metacommunities of the 174 nematode species included in this analysis mostly had a coherent pattern. The degree of turnover increased with increasing scale. Ordination scores correlated with geographical variables on the larger scales and with the trophic state index on a regional scale. The association of the structuring gradient with spatial variables and the scale-dependent increase in turnover showed that nematode dispersal was limited. The different metacommunity patterns identified at the increasing geographical scales suggested different, scale-related mechanisms of species distribution, with species sorting dominating on smaller and mass effects on larger geographical scales.

## Introduction

A primary goal of ecology is to measure, understand, and predict patterns of biodiversity [1], which in turn requires investigation of the factors that structure ecological communities [2]. These include not only environmental features, which shape species composition patterns, but also the connectivity of communities by dispersal, which influences species structure [3–5]. It is therefore important to consider how communities are connected and at which spatial scale [6, 7], given that the pattern may vary depending on the scale of observation [8, 9]

These considerations have given rise to the concept of metacommunity, defined as “a set of ecological communities at different sites, potentially but not necessarily linked by dispersal” [10]. The term has been applied in studies of the mechanisms underlying the dispersal of organisms, including within the context of community structure [11]. Four community structuring mechanisms have been proposed [9]: patch dynamic, species sorting, mass effects, and neutral perspective. They differ in their prerequisites concerning the homogeneity of the relevant sites and species. In the species sorting mechanism, metacommunities are structured primarily by environmental conditions [12]; in this case, the dispersal ability of a species is important because it enables it to track environmental gradients [9]. By contrast, according to the mass effects mechanism, the influence of environmental features overlaps with that of spatial characteristics, because local populations are quantitatively modified by species dispersal [4, 12].

To what extent environmental features and dispersal abilities are responsible for community structuring is thus far unclear. Cottenie and DeMeester [13] found evidence that environmental features are the primary factors influencing the structure of Cladocera communities. Soininen et al. [3] concluded that dispersal was the most relevant factor structuring zooplankton communities. Differences in the predominating factors identified in the different studies ([4, 5, 14] and others) are probably due to differences in the particular characteristics of the observed organisms [15]. For example, organisms with a low dispersal-ability may show a spatial distribution whereas more vagile species may be structured primarily by environmental conditions [2, 4, 12, 16].

Among the various methods used to examine metacommunity structure, that of Leibold and Mikkelson [10] focuses on species distribution patterns while ignoring species abundances, such that only the presence or absence of a species at a particular site is taken into account. This approach is based on the evaluation of three elements of metacommunity structure (EMS): coherence, turnover, and boundary clumping. A metacommunity is coherent if the majority of species colonize a coherent range of sites ordered according to a latent gradient [17]. The degree of turnover indicates the tendency of species to replace one another at a site. The inability of two species with different but coherent ranges to replace each other requires that they exhibit a nested pattern; hence, turnover can be used to measure nestedness [10]. Finally, boundary clumping evaluates whether the boundaries of species’ ranges coincide more or less than would be expected if they were established by chance; it therefore considers whether species respond to a latent gradient in the same way or not.

Lakes are suitable model systems to investigate metacommunities. As “islands” of freshwater organisms, they allow local communities to be readily distinguished [18]. Lakes vary in their size, environmental properties, and degree of isolation, which facilitates studies of the influences of these features on organismal distribution [2, 19]. However, studies on the mechanisms structuring metacommunities [19] have largely ignored freshwater meiofauna (but see [20, 21]). The most abundant and diverse meiobenthic freshwater taxon is formed by nematodes [22, 23]. Nematodes differ in their feeding type, which in turn determines the trophic level at which they are found. They can be classified as bacterial, algal, plant, or omnivorous feeders or as predators [24–26]. Nematodes can move actively over short distances or they may drift within a body of water [22, 27]. Their long-distance distribution depends on transport by vectors such as wind, rain, and other animals [28, 29] but it is also favored by several features inherent to the group Nematoda, including parthenogenesis, hermaphroditism, drought-resistant stages, anhydrobiosis and short generation times [22, 30–32]. Dispersal is also supported by the small size of nematodes, as dispersal potential increases with decreasing propagule size [16, 33, 34]. Thus, according to Fenchel and Finlay [35], organisms < 1 mm in length are likely to have a cosmopolitan distribution.

The scarcity of studies on nematode metacommunity structure [21] despite the ecological relevance of nematodes stimulated our interest in identifying their distributional patterns. We therefore analyzed the metacommunity structures of benthic nematodes in the littoral zones of 16 European lakes at four different geographical scales, ranging from 80 m to 360 km. Our hypotheses were: (1) Metacommunities of nematodes show a coherent pattern on every geographical scale. (2) The distribution properties of nematodes allow them to track changes in the features of their environments. Hence the effects of species sorting processes would be more apparent and the influence of site conditions on metacommunity structure would be paramount. We therefore further hypothesized that the structuring gradient of nematode-metacommunities correlates more strongly with the degree of eutrophication than with spatial components. (3) In addition, concerning the metric turnover, we assumed that the connectivity of communities on local scales (especially within lakes) leads to a nested pattern whereas passive dispersal at larger scales should result in a higher degree of turnover.

## Material and Methods

### Sampling sites

**Fig 1:**
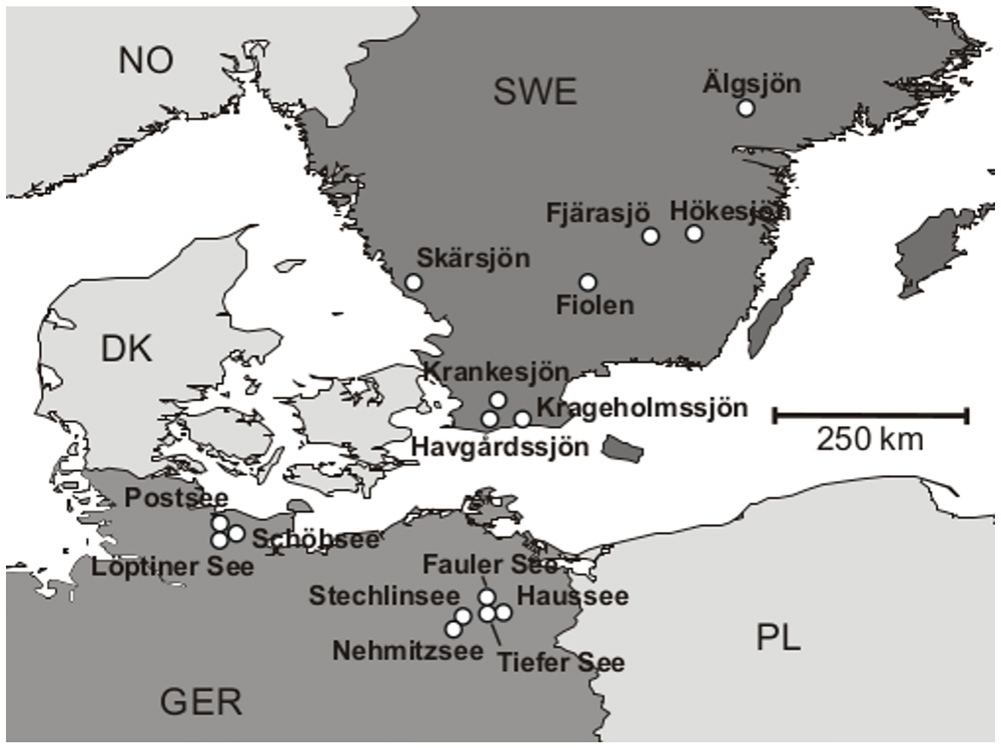
Locations of the sampled lakes.

We investigated the nematode communities in the littoral zones of 16 European lakes covering a gradient from oligotrophic to eutrophic conditions (Fig 1). Therefore data from a study of Ristau et al. [36] were supplemented by data on Lake Löptin (Löptiner See). Eight lakes were situated in southern Sweden and eight in northern Germany. Trophic state was evaluated using the trophic state index (TSI), following the method of Carlson [37]. The lakes were classified as oligotrophic (TSI ≤ 40), mesotrophic (40 < TSI ≤ 50), or eutrophic (TSI > 50) based on the total phosphorus and chlorophyll a concentration. These data were kindly provided by Swedish and German environmental agencies, with the exception of the Fauler See, where own measurements were made [36]. The number of sampling sites per lake varied between five and eight (three replicates per site), according to the accessibility of their littoral areas. The geographical data for all 16 lakes are provided in Table 1.

**Table 1:** Geographical and trophic data on the studied Swedish and German lakes. Trophic state index (TSI) was calculated according to the method of Carlson [37]. Data from a study of Ristau et al. [36] were supplemented by data on Lake Löptin (Löptiner See). Three replicates per sampling site.

Lake	Sampling sites (n)	Distance of two closest sites (m)	Latitude	Longitude	Elevation(m)	TSI	Trophic state
**Sweden**							
Havgardssjön	7	140	55°28'57.06"N	13°21'25.20"E	63	58.6	E
Krageholmssjön	6	50	55°30'0.82"N	13°44'45.01"E	41	65.4	E
Fiolen	8	50	57° 4'57.65"N	14°31'53.48"E	247	39.7	O
Skärsjön	8	50	57° 4'48.97"N	12°29'48.02"E	83	36.6	O
Krankesjön	6	100	55°42'2.60"N	13°28'40.39"E	16	53.1	E
Hökesjön	7	60	57°38'41.59"N	15°45'31.87"E	144	32.9	O
Fjärasjö	7	60	57°36'23.05"N	15°15'22.54"E	236	35.4	O
Älgsjön	5	50	59° 5'58.67"N	16°22'18.79"E	85	49.1	M
**Germany**							
Stechlinsee	7	80	53° 9'3.62"N	13° 1'38.50"E	65	41.2	M
Nehmitzsee	7	50	53° 7'40.04"N	12°58'56.66"E	58	48.6	M
Haussee	8	50	53°14'58.24"N	13°32'27.10"E	68	47.8	M
Fauler See	4	100	53°13'52.72"N	13°22'6.42"E	41	48.6	M
Tiefer See	5	160	53°14'7.35"N	13°21'42.68"E	62	46.0	M
Postsee	7	60	54°13'8.22"N	10°13'44.78"E	18	71.3	E
Schöhsee	8	80	54° 9'53.56"N	10°26'25.46"E	24	46.1	M
Löptiner See	5	50	54°10'33.54"N	10°13'15.87"E	24	78.4	E

O, oligotrophic; M, mesotrophic; E, eutrophic.

### Sampling

Sediment samples were collected either during spring 2008 or 2007. The uppermost 3 cm of the sediment layer was collected at a water depth of 0.4–0.6 m using an acrylic tube (internal diameter of 2.5 cm) and directly preserved in formaldehyde (final concentration 4%). The meiobenthic organisms were stained with Rose Bengal. Sediment and meiofaunal organisms were separated by density gradient centrifugation in colloidal silica (LUDOX TM 50: density adjusted to 1.14 g ml^−1^, mesh size 35 μm), as described by Pfannkuche and Thiel [38]. When present, the first 30 nematodes per replicate were processed for species identification according to the method of Seinhorst [39, 40]. In around 90% of all samples there were at least 25 individuals available forIdentification to the species level if possible (1000× magnification, oil immersion). 333 to 720 nematodes per lake were identified, in total nearly 9000 individuals.

No specific permission was required for these lakes and no protected species were sampled.

### Scales of analysis

The metacommunity structure was analyzed on the following four geographical scales:

1. *Local*: Three replicates were obtained from the two sites of each lake that were closest to each other (radius of analysis: max. 80 m, see Table 1).
2. *Lake*: Five to eight sites within each lake were compared and the data from the three replicates at each site were pooled.
3. *Regional*: The eight lakes of each country were compared separately and the data of all replicates per lake were pooled (radius of analysis: approx. 120 km in Germany and 160 km in Sweden).
4. *Supra-regional*: All 16 lakes were compared and the data from all replicates obtained from each lake were pooled (radius of analysis: approx. 360 km).

### Statistical analysis

The metacommunity structure analysis developed by Leibold and Mikkelson [10] was used to determine the best-fitting pattern of nematode species distribution. The data set was therefore arranged in presence/absence matrices, listing species in rows and communities in columns. In these matrices, a “1” in a cell *(i*,*j)* indicated the presence of species *i* at site *j*.

The matrices were then reordered using reciprocal averaging, which ranked the rows and columns such that those species (rows) with the closest distributions were located close to each other in the matrix, as were communities (columns) with the most similar lists of species [17]. According to Leibold and Mikkelson [10], a significantly coherent matrix is required to test turnover and boundary clumping. However, Dallas and Drake [2] cautioned against interpreting a non-significant result as evidence that a community is randomly structured. Therefore, the matrices tested for turnover and boundary clumping were those more likely to be coherent (p < 0.5).

Coherence was evaluated by counting the number of embedded absences in each of the ordinated matrices. An embedded absence is an interruption of the positive results (= 1) in a row or column [10] of the presence/absence matrix. To test the significance of the interruption, we used a z-test to compare the given number of embedded absences with the mean number obtained from 200 randomly generated and re-ordered matrices. According to the approach of Fontaneto et al. [20], these matrices had to fulfil the following conditions: (1) the number of “1” values in the null model is the same as in the original presence/absence matrix and (2) in each row and column there is at least one “1.”

To evaluate the amount of species turnover, we counted the number of times species replaced another between two sites. To avoid registering a correlation between replacements and embedded absences, the embedded absences in each row were filled-in before the number of replacements was counted. This number of replacements was also tested against the mean number determined in 200 randomly generated matrices. However, in this case, because the focus was on the pattern between rows, we used an algorithm that maintains the row sums but spread the “1” values randomly over the given set of sites.

To determine whether the species boundaries in a metacommunity coincide to a greater or lesser degree than expected by chance, we used Morisita’s index [41], according to the method described by Hoagland and Collins ([42] see formula on p. 26), to evaluate the clumping of species boundaries. Boundaries randomly distributed across a given set of sites have a Morisita’s index of 1. Values < 1 indicate that the mean range of boundaries is less clumped than expected by chance, whereas for values > 1 the boundaries are more clumped than in the null model.

As the distances of the sites at local scale varied, we checked if this distance has influence in coherence and turnover orientation using a Mann-Whitney-U-Test.

All statistical analyses of the metacommunity pattern were performed using the *R* package ‘metacom’ [11]. After determination of the EMS, the data were evaluated with respect to the different patterns of distribution following the approach of [17].

A by-product of the ordination procedure is that it determines site scores, which are used to order species and sites in the presence/absence matrix. These scores represent the structuring gradient and can be related to different variables [43, 44]. Significant correlations between site scores and geographical characteristics (longitude and latitude) were identified by calculating Spearman rank correlations using *SigmaPlot* (version 11.0). Testing was conducted on every scale except the local one, because exact coordinates were not available for each replicate. On the regional and supra-regional scales, the correlations between site scores and both elevation and the TSI were also investigated.

## Results

### Local scale

An average of 23.25 (± 7.33 SD) species per lake were identified. Except for the matrix representing Lake Krageholmsjön, the matrices of all lakes contained fewer embedded absences than would be expected by the null model (p < 0.5). In addition, eight of the 16 metacommunities exhibited a significantly coherent pattern (Table 2). The 15 matrices were tested for turnover and boundary clumping. A negative turnover, i.e., fewer replacements than expected based on the null model, was determined in nine of them. However, the difference was significant only in the matrix of the Haussee (p < 0.05). Analyses of boundary clumping yielded Morisita’s index ranging from values under 0.01 to 2.53. In the EMS analysis, half of the metacommunities were classified as random, four as quasi-nested, three as quasi-Gleasonian, and one as quasi-evenly spaced (Table 2).

**Table 2:** Results of the EMS analysis of nematodes in 16 European lakes at four different geographical scales. Metacommunity patterns were identified according to the method of Presley et al. [17]. Coherence and turnover were calculated by determining the number of embedded absences and replacements in the interaction matrix vs. the null distribution. Significant values are shown in bold face. N.A., not available.

Lake			Actual values		Coherence			Turnover				Clumping		Metacommunity pattern
	Species	Sites	EmbAbs	Rep	Mean	SD	p	Mean	SD	Orientation	p	I	p	
**Local scale**														
Havgardssjön	26	6	25	240	36.64	5.28	**0.028**	358.66	85.26	Negative	0.164	1.07	0.426	Quasi-nested (RSL)
Krageholmssjön	23	6	31	342	29.97	5.49	0.850	N.A.	N.A.	N.A.	N.A.	N.A.	N.A.	Random
Fiolen	38	6	47	917	59.43	8.72	0.154	975.12	204.52	Negative	0.776	0.00	0.134	Random
Skärsjön	34	6	39	965	48.81	8.93	0.272	831.17	180.99	Positive	0.460	0.00	0.116	Random
Krankesjön	22	6	17	396	28.98	5.25	**0.023**	339.59	80.85	Positive	0.485	0.00	**0.047**	Quasi-evenly spaced
Hökesjön	22	6	18	216	28.76	4.35	**0.013**	237.78	52.17	Negative	0.676	1.77	0.130	Quasi-nested (RSL)
Fjärasjö	21	6	20	180	27.15	4.64	0.123	254.21	63.77	Negative	0.245	0.84	0.449	Random
Älgsjön	28	6	22	371	39.07	6.68	**0.011**	487.66	112.99	Negative	0.302	0.58	0.319	Quasi-nested (RSL)
Stechlinsee	20	6	21	125	25.26	4.18	0.307	212.30	50.32	Negative	0.083	1.60	0.170	Random
Nehmitzsee	28	6	28	558	40.74	6.10	**0.037**	512.89	118.16	Positive	0.703	0.58	0.318	Quasi-Gleasonian
Haussee	11	6	8	26	10.47	2.80	0.378	62.75	18.63	Negative	**0.048**	2.00	**0.030**	Random
Fauler See	21	6	19	306	25.33	5.23	0.226	293.05	65.39	Positive	0.843	2.53	**0.023**	Random
Tiefer See	31	6	38	644	43.67	7.48	0.449	650.23	148.20	Negative	0.966	0.64	0.361	Random
Postsee	16	6	11	90	18.71	3.78	**0.041**	123.46	32.75	Negative	0.307	2.49	**0.015**	Quasi-nested (CSL)
Schöhsee	18	6	11	160	21.80	4.13	**0.009**	140.35	38.85	Positive	0.613	1.42	0.222	Quasi-Gleasonian
Löptiner See	13	6	7	96	13.49	2.72	**0.017**	87.92	25.47	Positive	0.874	0.49	0.137	Quasi-Gleasonian
**Lake scale**														
Havgardssjön	42	7	59	1405	89.67	8.56	**<0.001**	1106.30	249.39	Positive	0.231	0.00	0.095	Quasi-Gleasonian
Krageholmssjön	30	6	22	384	40.97	5.13	**<0.001**	332.07	79.99	Positive	0.516	0.62	0.347	Quasi-Gleasonian
Fiolen	77	8	208	4107	232.21	17.48	0.166	4029.42	926.44	Positive	0.933	2.47	0.065	Random
Skärsjön	74	8	183	4022	220.99	18.63	**0.041**	4149.40	938.19	Negative	0.892	0.79	0.430	Quasi-nested (RSL)
Krankesjön	36	6	53	483	55.09	6.66	0.750	N.A	N.A	N.A.	N.A.	N.A.	N.A.	Random
Hökesjön	40	7	38	742	81.84	7.90	**<0.001**	849.05	198.27	Negative	0.589	0.58	0.313	Quasi-nested (RSL)
Fjärasjö	50	7	79	1103	108.42	8.20	**<0.001**	1288.95	285.99	Negative	0.516	0.00	0.120	Quasi-nested (RSL)
Älgsjön	43	5	35	805	47.19	6.66	0.067	769.79	150.90	Positive	0.815	0.00	0.225	Random
Stechlinsee	36	7	40	892	72.80	8.13	**<0.001**	728.74	183.90	Positive	0.375	0.30	0.450	Quasi-Gleasonian
Nehmitzsee	39	7	71	930	78.38	6.94	0.290	843.62	184.66	Positive	0.640	0.56	0.302	Random
Haussee	30	8	57	808	73.17	7.64	**0.030**	714.58	155.98	Positive	0.540	0.31	0.096	Quasi-Gleasonian
Fauler See	35	4	12	488	20.59	3.53	**0.015**	412.02	59.05	Positive	0.198	0.00	0.282	Quasi-Gleasonian
Tiefer See	54	5	48	1645	63.80	8.81	0.073	1333.32	284.13	Positive	0.273	0.00	0.254	Random
Postsee	22	7	25	181	36.36	4.96	**0.022**	220.45	51.47	Negative	0.443	0.91	0.477	Quasi-nested (RSL)
Schöhsee	31	8	71	702	76.05	6.91	0.470	679.45	156.19	Positive	0.885	0.32	0.100	Random
Löptiner See	15	5	8	80	11.21	2.75	0.240	65.20	18.31	Positive	0.419	0.46	0.234	Random
**Regional scale**														
Swedish lakes	137	8	292	12788	440.76	23.63	**<0.001**	10800.27	2409.53	Positive	0.409	0.00	0.218	Quasi-Gleasonian
German lakes	92	8	177	3720	284.34	18.04	**<0.001**	5021.42	1315.02	Negative	0.322	0.00	0.165	Quasi-nested (RSL)
**Supra-regional scale**														
All lakes	174	16	938	69612	1604.74	51.24	**<0.001**	40426.72	13276.28	Positive	**0.028**	0.79	0.384	Gleasonian

RSL, Random species loss; CSL, Clumped species loss

EmbAbs, Number of embedded absences; Rep, Number of replacements; I, Morisita’s index

Furthermore the distance between the two sites used for the analysis on local scale had neither a significant influence on coherence (p = 0.87) nor on turnover orientation (p = 0.85) and therefore did not affect the metacommunity pattern.

### Lake scale

Analyses of the species present in the lake samples revealed a mean of 40.88 (±16.55) species per lake. The largest species diversity was found in Lake Fiolen (77 species) whereas very few species (15) were detected in Löptiner See. All matrices showed fewer embedded absences than expected on the basis of the null model (Table 2). Nine metacommunities had a significantly coherent pattern. Among the 15 matrices tested for turnover and boundary clumping, four of the respective metacommunities had a negative turnover; in the remaining 11 the turnover was positive but in none of them was the difference between the actual and expected number of replacements significant. Except for Lake Fiolen, all tested lakes showed a less clumped pattern than expected by chance but none of the patterns were significant. Seven metacommunites were characterized as random, five as quasi-Gleasonian, and four as quasi-nested.

### Regional scale

In the Swedish lakes 137 nematode species were detected and in the German lakes 92 species. In the matrices of both sets of lakes, the number of embedded absences was significantly (p < 0.001) less than predicted by the null model, which indicated the coherence of the metacommunities of German and of Swedish lakes. The number of replacements was greater than expected in the matrix of the Swedish lakes and fewer than expected in that of the German lakes. Consequently, metacommunity turnover was positive in the Swedish lakes and negative in the German lakes. Morisita’s index was < 1 in both matrices. The turnover in the metacommunity of the Swedish lakes was quasi-Gleasonian whereas in the German lakes it was quasi-nested.

### Supra-regional scale

Among the 174 nematode species identified in this study (see list of species, S1 Table), 58 occurred in Swedish and in German lakes. Four occurred in all 16 lakes: *Eumonhystera vulgaris*, *Prodesmodora circulata*, *Tobrilus gracilis*, and *Tripyla glomerans*. 35 species were found only in the German lakes and 81 species only in the Swedish lakes.

The metacommunity across all lakes was significantly (p < 0.001) coherent. The number of replacements was significantly (p < 0.05) higher than expected, indicating a positive turnover. Together with a Morisita’s index of 0.79, these values indicated the Gleasonian pattern of the metacommunity.

### Correlation with site scores

The coordinates of several sampling sites of a lake did not generally correlate with the site scores of the respective presence/absence matrices. The exception was Lake Fjärasjö, where the site scores were highly correlated with latitude (correlation coefficient (r) = 0.93, p < 0.001).

On the regional scale the matrix values of the German lakes correlated with both latitude (r = −0.69, p < 0.05) and longitude (r = 0.69, p < 0.05). By contrast, there were no correlations with the site scores of the Swedish lakes, although a Spearman test revealed a correlation between site scores and the TSI (r = −0.76, p = 0.021) and between site scores and elevation (r = 0.81, p = 0.01).

The site scores on the supra-regional scale correlated with latitude (r = 0.72, p <0.001), longitude (r = 0.54, p = 0.03), and elevation (r = 0.62, p = 0.01) but not with the degree of eutrophication.

## Discussion

This is one of the first studies to evaluate a wide spatial range in a metacommunity analysis of nematodes. It provides insight into metacommunity patterns and the possible factors that structure them.

All of the presence/absence matrices except one showed a coherent pattern as they had fewer embedded absences than expected. In 20 of the 35 matrices, the difference was significant. These results support hypothesis (1), that nematode metacommunities show a coherent pattern on every geographical scale considered. Therefore, nematodes in a metacommunity are apparently able to respond to the same latent gradient. The importance of this finding is that it enables exploration of the gradients that induce metacommunity patterning and provides a plausible interpretation of the measurements of turnover and boundary clumping [10].

There are several reasons for the lack of significant coherence in some matrices. The first is statistical: because the number of sites and species increased with increasing geographical scale, the tests carried out for larger-scale areas were statistically more meaningful. Second, several species were found only at one sampling site, which resulted in gaps in the rows and columns of the matrices and therefore affect numbers of embedded absences [44, 45]. The weak coherence suggested that not all of the nematode species responded to the same environmental gradient but instead were partially associated with different gradients [17]. This may have been a consequence of the various nematode feeding types (see S1 Table), which would result in different ecological dependencies.

Further insights into the characteristics of the structuring gradient were obtained by testing the site scores from the reciprocal averaging for correlations with geographical variables and the eutrophic degree (TSI). However, the method used to characterize the structuring gradient of metacommunities does not yield information on the relative influence of single variables [2] and thus provides only a first impression. Accordingly, hypothesis (2), that the distribution properties of nematodes enable these organisms to track changes in the features of their environments, may not be valid in general. On a per-lake scale, correlation tests were possible only to a limited extent, as TSI data were not available for single sampling sites. Although it was therefore not possible to draw conclusions regarding the influence of the degree of eutrophication on metacommunity structure, we were able to show that spatial parameters were not associated with the primary structuring gradient on this smaller scale of analysis. By contrast, on the regional scale the site scores correlated with the latitude and longitude of German lakes and on the supra-regional scale with all geographical variables. These results are in accordance with the data of Flach et al. [46] and Zullini [47], who demonstrated the spatial structure of nematode communities at larger geographical scales.

Site scores correlated with the TSI only on the regional scale of the Swedish lakes. Ristau and Traunspurger [48] used the same dataset but a different statistical analysis to show that the degree of eutrophication is a relevant factor in the structuring of communities. The consistency of these findings and our own supports the informative value of the EMS analysis.

In addition, our results evidenced a correlation between the structuring gradient and elevation, both on a regional (Swedish lakes) and a supra-regional scale. Other studies have similarly identified a relation between elevation and species assemblages [49, 50]. Lake elevation probably affects either the environmental conditions [51] or the passive dispersal of nematodes. Overall, in contrast to the regional-scale results obtained for German lakes, the structuring gradient of Swedish lakes was more closely associated with environmental conditions than with spatial components. This may have been due to the wider range of eutrophic states and altitudes covered by the Swedish lakes (Table 1).

In summary, variables correlating with the structuring gradient changed over the different scales of analysis whereas the influence of spatial components increased progressively. This outcome suggests that different mechanisms of dispersal predominate at different geographical scales. While the dominance of environmental features indicated species sorting effects [12, 15], the spatial structure of the analyzed species was consistent with mass effects [4, 12]. The varying mechanism may reflect the different modes of nematode dispersal [1, 4]. Previous studies showed that passive dispersal may result in spatially structured patterns [4, 52], consistent with our findings for nematodes on the supra-regional scale. This assumption is relevant only as long as dispersal rates are not high enough to result in “everything is everywhere” [35], which would lead to a system dominated by species sorting processes. Thus, it can be hypothesized that on small geographical scales the rates of nematode dispersal are high, because of species sorting effects, while on larger scales barriers to dispersal result in a spatial community structure shaped by mass effects.

In many cases, the distributional pattern seemed to be a random one, according to the framework of Presley et al. [17], although coherence and turnover revealed interesting results. Thus, in the remainder of this discussion we assess the individual metrics rather than the identified types of metacommunity patterns.

Because only one significantly nested pattern was detected (Haussee local scale, Table 2), the results of the turnover analysis did not clearly confirm hypothesis (3): the connectivity of communities on local scales leads to a nested pattern whereas passive dispersal at larger scales result in a higher degree of turnover. Nevertheless, there was an increasing tendency of positive turnover; that is, species replaced one another between two sites more often than would have been expected by chance. Turnover on the local scale was positive in six matrices, on the lake scale in 11 matrices, on the regional scale of the Swedish lakes, and on the supra-regional scale (Table 2). The increase in turnover with increasing scale suggests a more diverse species composition over larger geographical scales. This is a plausible result, assuming that nematodes are subject to dispersal limitations over larger distances, as reported by Ptatscheck et al. [21].

Interestingly, the results of the turnover analysis differed on the regional scale, that is, between the German and Swedish lakes. This can be explained by the aggregated locations of the German lakes whereas the Swedish lakes are distributed more evenly over the southern part of the country (Fig 1). The negative turnover in the matrix of German lakes could have been due to adjustments in species composition in nearby lakes. This hypothesis is supported by the fact that lakes with an aggregated location occupied adjacent positions in the ordinated matrix and therefore had closely related species compositions.

In general the matrices showed a random boundary clumping. Hence no group of species could be detected to respond synchronously on the structuring gradient.

## Conclusion

The results of our EMS analysis of nematodes largely agreed with those of other studies, which recommends this approach in further investigations based on nematode abundance data. Specifically, we found that nematodes, despite their small size and potential for dispersal, may in general not have a cosmopolitan distribution, which implies limits to their long-distance dispersal. Metacommunity patterns were shown to vary on different geographical scales and different variables were found to be associated with the structuring gradient. Taken together, our findings suggest that the mechanisms structuring nematode metacommunity patterns change, from species sorting on small spatial scales to mass effects on larger ones.

## Supporting Information

S1 Fig Presence/ absence matrices for each scale of analysis. (PDF)

S1 Table Nematode species found in the 16 sampled lakes in Sweden and Germany. (DOCX)
