# Supplementary material for: Varying Patterns on Varying Scales: A Metacommunity Analysis of Nematodes in European Lakes
Source: PLoS One. 2016 Mar 23;11(3):e0151866. doi: 10.1371/journal.pone.0151866 (PMC4805234; doi:10.1371/journal.pone.0151866)
Supplement: S1 Fig — (PDF) [file pone.0151866.s001.pdf]

a) Havgardssjön

Sites (columns) × *Species* (rows)

1	0	0	0	0	0
1	0	0	0	0	0
1	0	0	0	0	0
1	1	0	1	0	0
0	0	1	0	0	0
1	1	1	1	0	1
1	1	1	0	1	1
1	1	1	1	1	1
1	1	1	1	1	1
0	1	1	1	1	0
1	0	1	1	1	1
1	0	0	1	0	1
0	0	0	1	0	0
0	0	0	1	0	0
0	1	1	1	1	1
0	0	0	0	1	0
0	0	0	1	0	1
0	0	0	1	0	1
0	0	0	1	0	1
0	0	0	0	1	1
0	0	0	0	1	1
0	0	0	0	0	1
0	0	0	0	0	1
0	0	0	0	0	1
0	0	0	0	0	1
0	0	0	0	0	1

b) Krageholmssjön

Sites (columns) × *Species* (rows)

1	0	0	0	0	0
1	0	0	0	0	0
1	0	0	0	0	0
1	1	1	0	0	0
1	0	1	1	0	0
1	1	0	1	1	0
1	1	1	1	1	1
0	1	0	1	0	0
0	0	1	0	0	0
0	0	1	0	0	0
0	0	1	0	0	0
0	0	0	1	0	0
0	1	1	0	1	0
0	1	1	1	0	1
0	1	1	0	0	1
0	0	0	1	1	0
0	0	1	1	0	1
0	0	1	1	1	1
0	0	0	0	1	0
0	0	0	0	1	0
0	0	0	0	1	1
0	0	0	0	0	1
0	0	0	0	0	1

c) Fiolen

Sites (columns) × *Species* (rows)

1	0	0	0	0	0
1	0	0	0	0	0
1	0	0	0	0	0
1	1	0	0	0	0
1	1	0	0	0	0
1	1	0	0	0	0
1	1	0	0	0	0
0	1	0	0	0	0
0	1	0	0	0	0
0	1	0	0	0	0
0	1	0	0	0	0
1	1	1	0	0	0
1	1	1	0	0	0
1	1	0	1	0	0
1	1	0	0	0	1
1	1	0	1	0	1
1	0	0	0	1	0
0	1	0	0	0	1
1	1	0	1	1	1
0	1	1	0	1	0
1	0	1	0	0	1
0	1	1	0	0	1
0	1	1	1	1	0
1	0	0	0	1	1
0	0	1	0	0	0
0	0	1	0	0	0
0	0	1	0	1	0
0	0	0	1	0	0
0	0	1	0	0	1
0	0	1	1	1	1
0	0	0	1	1	0
0	0	0	1	1	1
0	0	0	0	1	1
0	0	0	0	0	1
0	0	0	0	0	1
0	0	0	0	0	1
0	0	0	0	0	1
0	0	0	0	0	1

d) Skärsjön

Sites (columns) × *Species* (rows)

1	0	0	0	0	0
1	0	0	0	0	0
1	1	0	0	0	0
1	1	0	0	0	0
0	1	0	0	0	0
0	1	0	0	0	0
1	1	1	0	0	0
1	0	1	0	0	0
1	0	0	1	0	0
0	0	1	0	0	0
0	0	1	0	0	0
0	0	1	0	0	0
0	1	0	0	1	0
1	1	1	1	1	1
0	0	1	1	0	0
1	1	0	1	1	1
0	0	0	1	0	0
0	0	0	1	0	0
0	1	1	1	1	1
0	0	0	1	1	0
0	0	0	1	1	0
0	0	1	1	1	1
0	0	1	0	1	1
0	0	0	0	1	0
0	0	0	0	1	0
0	0	0	0	1	0
0	0	0	0	1	0
0	0	0	0	1	1
0	0	0	0	0	1
0	0	0	0	0	1
0	0	0	0	0	1
0	0	0	0	0	1
0	0	0	0	0	1
0	0	0	0	0	1

e) Krankesjön

Sites (columns) × *Species* (rows)

1	0	0	0	0	0
1	0	0	0	0	0
1	0	0	0	0	0
1	0	0	0	0	0
1	1	0	0	0	0
0	1	0	0	0	0
1	1	1	0	0	0
1	1	1	0	0	0
1	1	1	1	0	0
1	0	0	0	0	1
1	1	1	1	1	0
1	1	1	1	1	1
0	1	0	0	0	1
0	0	1	0	0	1
0	0	1	0	0	1
0	0	0	1	0	0
0	0	1	0	1	1
0	0	0	1	1	1
0	0	0	1	1	1
0	0	0	0	1	1
0	0	0	0	0	1
0	0	0	0	0	1

f) Algsjön

Sites (columns) × *Species* (rows)

1	0	0	0	0	0
1	0	0	0	0	0
1	0	0	0	0	0
1	0	0	0	0	0
1	1	1	0	0	0
1	1	1	1	0	0
1	1	1	0	0	1
0	0	1	0	0	0
0	0	1	0	0	0
1	0	1	1	1	0
1	1	1	1	1	1
0	1	1	1	1	1
0	0	1	0	1	1
0	0	1	1	1	1
0	0	0	1	0	0
0	0	0	1	0	0
0	0	0	1	0	1
0	0	0	1	0	1
0	0	0	1	1	1
0	0	0	1	1	1
0	0	0	0	1	0
0	0	0	0	1	0
0	0	0	0	1	0
0	0	0	0	1	1
0	0	0	0	0	1
0	0	0	0	0	1
0	0	0	0	0	1
0	0	0	0	0	1

g) Fjärasjö

Sites (columns) × *Species* (rows)

1	0	0	0	0	0
1	0	0	0	0	0
1	0	0	0	0	0
1	1	0	0	0	0
1	1	0	0	0	0
1	1	1	0	1	0
1	1	0	1	0	1
1	1	0	1	0	1
1	1	1	1	1	0
0	1	0	1	0	0
0	1	1	1	0	0
1	0	1	1	0	1
1	1	1	1	1	1
0	1	0	1	0	1
0	0	1	1	0	1
0	0	1	1	1	1
0	0	0	1	0	1
0	0	0	1	0	1
0	0	0	0	1	1
0	0	0	0	1	1
0	0	0	0	0	1

h) Hökesjön

Sites (columns) × *Species* (rows)

1	0	0	0	0	0
1	0	0	0	0	0
1	0	0	0	0	0
1	0	0	0	0	0
1	0	0	0	0	0
1	1	1	1	0	0
1	1	0	0	1	1
1	1	0	0	1	1
1	1	1	1	0	1
1	0	1	1	1	1
1	1	1	1	1	1
1	1	1	1	1	1
0	1	1	1	1	0
0	1	0	0	0	1
0	1	0	1	0	1
0	0	1	0	1	1
0	0	0	1	0	0
0	0	0	0	1	0
0	0	0	1	1	1
0	0	0	1	0	1
0	0	0	0	1	1
0	0	0	0	0	1

S1A Figure. Presence/ absence matrices of Swedish lakes on local scale. Matrices ordinated using reciprocal averaging.

a) Stechlinsee

Sites / *Species*

1	0	0	0	0	0
1	0	0	0	0	0
1	0	0	0	0	0
1	0	0	0	0	0
1	0	0	0	0	0
1	1	0	0	0	1
1	0	1	1	1	0
1	0	1	1	0	1
1	0	1	0	1	1
1	1	1	1	0	1
1	1	1	1	1	1
1	1	1	1	1	1
0	1	0	0	0	0
0	1	1	0	1	0
0	0	1	0	0	0
0	0	1	1	0	1
0	0	0	1	0	1
0	0	0	0	1	1
0	0	0	0	0	1
0	0	0	0	0	1

b) Nehmitzsee

Sites / *Species*

1	0	0	0	0	0
1	0	0	0	0	0
1	0	0	0	0	0
1	0	0	0	0	0
1	0	0	0	0	0
1	1	0	0	0	0
1	0	1	0	0	0
1	0	1	0	0	0
0	0	1	0	0	0
0	0	1	0	0	0
1	0	1	1	0	1
1	0	1	0	1	1
1	0	0	1	1	0
1	1	1	1	1	1
1	1	1	1	1	1
1	1	1	1	1	1
0	1	0	0	1	0
0	0	1	0	1	0
0	0	1	0	0	1
0	0	1	0	1	1
0	0	0	1	0	0
0	0	0	1	0	0
0	0	0	1	0	1
0	0	0	1	1	1
0	0	0	0	1	0
0	0	0	0	1	0
0	0	0	0	1	1
0	0	0	0	0	1

c) Tiefer See

Sites / *Species*

1	0	0	0	0	0
1	0	0	0	0	0
1	0	0	0	0	0
1	0	0	0	0	0
1	0	0	0	0	0
1	0	0	0	0	0
1	0	0	1	0	0
1	1	1	0	0	0
0	1	0	0	0	0
0	1	0	0	0	0
0	1	0	0	0	0
1	0	0	1	0	1
1	1	1	0	1	1
1	1	1	1	1	1
1	0	0	1	1	1
0	0	1	0	0	0
0	0	1	0	0	0
0	1	1	1	1	1
0	0	0	1	0	0
0	0	1	1	0	1
0	0	0	1	1	0
0	0	0	1	1	0
0	0	1	0	1	1
0	0	0	1	0	1
0	0	0	1	0	1
0	0	0	0	1	0
0	0	0	0	1	1
0	0	0	0	0	1
0	0	0	0	0	1
0	0	0	0	0	1
0	0	0	0	0	1

d) Schöhsee

Sites / *Species*

1	0	0	0	0	0
1	0	0	0	0	0
1	0	0	0	0	0
1	1	0	0	0	0
1	0	0	1	0	0
0	1	0	0	0	0
0	1	0	0	0	0
1	1	1	0	1	1
1	1	1	0	1	1
1	1	1	1	1	1
1	1	1	1	1	1
1	0	1	1	1	1
0	1	1	1	1	1
0	0	0	1	1	1
0	0	0	0	1	0
0	0	0	0	1	0
0	0	0	0	0	1
0	0	0	0	0	1

e) Postsee

Sites / *Species*

1	0	0	0	0	0
1	0	0	0	0	0
1	1	0	0	0	0
0	1	0	0	0	0
1	1	1	1	1	1
1	1	1	1	1	1
1	1	1	1	1	1
1	0	1	1	0	1
1	0	1	1	0	1
0	1	0	1	0	1
0	0	0	1	0	0
0	0	0	0	1	0
0	0	0	1	1	1
0	0	0	0	0	1
0	0	0	0	0	1
0	0	0	0	0	1

f) Löptiner See

Sites / *Species*

1	0	0	0	0	0
1	1	0	0	0	0
1	1	1	0	0	0
0	1	0	0	0	0
1	1	0	0	0	1
1	1	1	1	1	0
1	1	1	1	1	1
1	1	1	1	1	1
0	1	0	1	1	1
0	0	0	1	1	1
0	0	0	1	1	1
0	0	0	0	1	1
0	0	0	0	0	1

g) Haussee

Sites / *Species*

1	0	0	0	0	0
1	1	1	1	1	1
1	1	1	1	1	1
0	1	1	1	1	0
0	1	0	0	0	1
0	0	0	1	0	0
0	0	1	1	1	1
0	0	0	1	1	1
0	0	0	0	1	0
0	0	0	0	1	1
0	0	0	0	0	1

h) Fauler See

Sites / *Species*

1	0	0	0	0	0
1	0	0	0	0	0
1	0	0	0	0	0
1	1	0	0	0	0
0	1	0	0	0	0
0	1	0	0	0	0
1	1	1	1	1	1
1	1	1	1	1	1
0	1	0	0	0	1
0	0	1	0	0	0
0	1	0	0	1	1
0	1	1	1	1	1
0	0	0	1	0	0
0	0	0	1	0	0
0	0	1	0	1	1
0	0	0	1	0	1
0	0	0	0	1	1
0	0	0	0	1	1
0	0	0	0	0	1
0	0	0	0	0	1
0	0	0	0	0	1

S1B Figure. Presence/ absence matrices of German lakes on local scale. Matrices ordinated using reciprocal averaging.

a) Havgardssjön

Sites (columns) × *Species* (rows)

1	0	0	0	0	0	0
1	1	0	0	0	0	0
1	1	0	0	0	0	0
0	1	0	0	0	0	0
0	1	1	0	0	0	0
0	1	1	0	0	0	0
0	1	0	1	0	0	0
1	1	1	1	0	1	0
0	0	1	0	0	0	0
0	0	1	0	0	0	0
0	0	1	0	0	0	0
0	0	1	0	0	0	0
1	1	1	0	1	1	0
1	1	1	1	0	1	1
0	1	0	1	0	1	0
1	1	0	1	0	1	1
1	1	1	1	1	1	1
1	1	1	1	1	1	1
0	1	1	1	0	1	1
1	0	1	1	1	1	1
0	1	1	0	0	1	1
0	1	1	1	1	1	1
0	1	1	0	1	1	1
0	0	1	1	0	1	1
0	0	0	1	0	0	1
0	0	1	0	0	1	1
0	0	1	0	1	1	1
0	0	0	0	1	0	0
0	0	0	0	1	0	0
0	0	0	0	0	1	0
0	0	0	0	0	1	0
0	0	0	0	0	1	0
0	0	0	0	0	1	0
0	0	0	0	0	1	0
0	0	0	0	0	1	0
0	0	0	0	1	1	1
0	0	0	0	0	1	1
0	0	0	0	0	1	1
0	0	0	0	0	0	1
0	0	0	0	0	0	1
0	0	0	0	0	0	1
0	0	0	0	0	0	1

b) Fjärasjö

Sites (columns) × *Species* (rows)

1	0	0	0	0	0	0
1	0	0	0	0	0	0
1	1	0	0	0	0	0
1	1	1	0	0	0	0
1	1	1	0	1	0	0
1	0	1	1	0	0	0
1	1	1	0	0	1	0
1	1	1	0	0	0	1
1	1	1	1	0	1	0
1	0	1	0	0	1	0
1	1	1	1	1	1	0
1	1	1	1	1	1	0
1	1	1	1	1	1	0
1	1	1	1	0	1	1
1	1	1	1	1	1	1
1	1	1	1	1	1	1
1	1	1	1	1	1	1
1	1	1	1	1	1	1
1	1	0	1	1	1	1
1	0	1	0	1	1	1
1	0	1	1	1	1	1
0	0	1	0	0	1	0
0	0	1	0	0	0	1
0	0	0	1	0	0	0
0	0	0	1	0	0	0
0	0	0	1	0	0	0
0	0	0	1	0	0	0
0	0	0	1	0	0	0
0	0	0	0	1	0	0
0	0	0	0	1	0	0
0	0	0	0	1	0	0
0	0	0	0	1	0	0
0	0	0	0	1	0	0
0	0	1	1	1	1	1
0	0	1	1	0	1	1
0	0	0	1	1	1	1
0	0	0	0	1	1	0
0	0	0	1	0	1	1
0	0	0	1	0	1	1
0	0	0	0	1	1	1
0	0	0	0	0	1	0
0	0	0	0	0	1	0
0	0	0	0	0	1	0
0	0	0	0	0	1	0
0	0	0	0	0	1	0
0	0	0	0	0	1	1
0	0	0	0	0	1	1
0	0	0	0	0	0	1
0	0	0	0	0	0	1

c) Fiolen

Sites (columns) × *Species* (rows)

1	0	0	0	0	0	0	0
1	0	0	0	0	0	0	0
1	0	0	0	0	0	0	0
1	0	0	0	0	0	0	0
1	0	0	0	0	0	0	0
1	0	0	0	0	0	0	0
1	0	0	0	0	0	0	0
1	1	0	0	0	0	0	0
1	1	0	0	0	0	0	0
1	1	1	0	0	0	0	0
1	1	1	0	0	0	0	0
0	1	0	0	0	0	0	0
0	1	0	0	0	0	0	0
1	1	0	1	0	0	0	0
1	1	0	1	0	0	0	0
1	0	0	1	0	0	0	0
1	1	0	0	1	0	0	0
1	0	0	0	1	0	0	0
1	1	1	1	0	0	0	0
1	1	1	0	1	0	0	0
1	1	1	0	1	0	0	0
1	1	1	0	1	0	0	0
1	1	1	0	0	1	0	0
0	0	1	0	0	0	0	0
0	0	1	0	0	0	0	0
0	0	1	0	0	0	0	0
0	0	1	0	0	0	0	0
1	1	1	0	1	1	0	0
1	1	0	0	0	0	0	1
0	1	1	1	0	0	0	0
0	1	1	0	1	0	0	0
0	1	1	0	0	1	0	0
0	1	1	0	0	1	0	0
0	1	0	1	0	0	0	0
0	1	0	1	0	0	0	0
0	1	0	0	1	0	0	0
1	1	0	1	0	0	0	1
0	1	1	1	1	0	0	0
1	1	0	0	0	1	0	1
0	0	1	1	0	0	0	0
1	1	1	1	1	1	0	1
1	1	1	1	1	1	0	1
1	1	0	0	1	1	0	1
1	0	1	0	1	1	0	1
1	1	1	0	1	1	1	1
1	0	1	1	1	1	0	1
1	0	1	1	1	1	0	1
1	0	1	1	1	1	0	1
0	0	1	1	0	1	0	0
0	1	1	1	1	1	1	0
0	1	0	1	0	0	1	0
0	0	0	1	0	0	0	0
0	1	1	1	1	1	1	1
0	0	0	0	1	0	0	0
0	0	0	0	1	0	0	0
0	0	0	0	1	0	0	0
0	0	0	0	1	0	0	0
0	0	0	0	1	0	0	0
0	0	0	0	1	0	0	0
0	0	0	0	1	0	0	0
0	0	1	1	0	0	0	1
0	1	0	1	1	1	1	1
0	0	0	0	0	1	0	0
0	0	0	0	0	1	0	0
0	0	0	0	0	1	0	0
0	0	0	0	0	1	0	0
0	0	0	0	0	1	0	0
0	1	0	1	0	1	1	1
0	0	0	1	1	0	0	1
0	0	0	1	0	0	0	1
0	0	0	1	0	0	0	1
0	0	0	0	1	0	0	1
0	0	0	0	1	0	0	1
0	0	0	0	0	0	0	1
0	0	0	0	0	0	0	1
0	0	0	0	0	0	0	1
0	0	0	0	0	0	0	1

d) Skärsjön

Sites (columns) × *Species* (rows)

1	0	0	0	0	0	0	0
1	0	0	0	0	0	0	0
1	0	0	0	0	0	0	0
1	0	0	0	0	0	0	0
1	0	0	0	0	0	0	0
1	0	0	0	0	0	0	0
1	0	0	0	0	0	0	0
1	0	0	0	0	0	0	0
1	1	0	0	0	0	0	0
1	1	0	0	0	0	0	0
1	0	1	0	0	0	0	0
1	0	1	0	0	0	0	0
1	0	1	0	0	0	0	0
1	0	1	0	0	0	0	0
1	1	1	0	0	0	0	0
0	1	0	0	0	0	0	0
0	1	0	0	0	0	0	0
0	1	0	0	0	0	0	0
0	1	0	0	0	0	0	0
0	1	0	0	0	0	0	0
0	1	0	0	0	0	0	0
0	1	0	0	0	0	0	0
1	1	0	0	0	1	0	0
1	0	0	1	0	0	0	0
1	1	1	1	0	0	0	0
1	0	0	0	1	0	0	0
1	1	1	0	0	1	0	0
1	0	1	0	1	0	0	0
1	0	1	0	0	1	0	0
1	1	0	0	0	0	0	1
1	1	1	0	0	0	1	0
1	0	1	1	1	0	0	0
0	0	1	0	0	0	0	0
0	0	1	0	0	0	0	0
0	0	1	0	0	0	0	0
1	1	1	0	1	0	1	0
1	0	1	0	0	0	0	1
0	1	1	1	1	0	0	0
1	0	0	0	0	0	0	1
1	1	0	1	0	1	0	1
0	1	0	0	1	1	0	0
0	0	1	0	1	0	0	0
0	0	1	0	1	0	0	0
0	1	0	1	1	1	0	0
1	0	0	1	1	0	1	0
0	0	1	0	0	1	0	0
1	1	1	1	0	1	1	1
1	0	0	1	0	1	1	0
1	1	0	1	1	1	1	1
0	1	1	1	1	1	1	0
0	0	0	1	0	0	0	0
0	0	0	1	0	0	0	0
0	0	0	1	0	0	0	0
0	0	0	0	1	0	0	0
0	1	1	1	1	1	1	1
0	0	1	1	1	0	1	0
0	0	1	1	1	1	1	0
0	0	1	0	1	1	1	0
0	1	0	1	1	1	1	1
0	0	1	0	0	0	1	0
0	0	1	0	0	1	1	0
0	0	0	0	0	1	0	0
0	0	1	0	0	0	0	1
0	0	0	0	1	1	1	1
0	0	0	0	0	1	1	0
0	0	0	0	0	0	1	0
0	0	0	0	0	0	1	0
0	0	0	0	0	0	1	0
0	0	0	0	0	0	1	0
0	0	0	0	0	0	1	1
0	0	0	0	0	0	0	1
0	0	0	0	0	0	0	1
0	0	0	0	0	0	0	1
0	0	0	0	0	0	0	1

e) Algsjön

Sites (columns) × *Species* (rows)

1	0	0	0	0
1	0	0	0	0
1	0	0	0	0
1	0	0	0	0
1	0	0	0	0
1	0	0	0	0
1	0	0	0	0
1	0	0	0	0
1	0	0	0	0
1	0	0	0	0
1	0	1	0	0
1	0	0	1	0
1	1	1	0	0
1	1	0	1	0
1	1	0	0	1
1	1	0	0	1
1	1	1	0	1
1	1	1	0	1
1	1	1	1	1
1	1	1	1	1
1	1	1	1	1
1	1	1	1	1
0	1	0	0	0
0	1	0	0	0
0	1	1	1	0
0	1	1	0	1
0	1	1	0	1
0	1	0	1	1
0	0	1	0	0
0	0	0	1	0
0	0	0	1	0
0	0	0	1	0
0	0	0	1	0
0	0	1	1	1
0	0	1	0	1
0	0	1	0	1
0	0	1	0	1
0	0	1	0	1
0	0	0	1	1
0	0	0	0	1
0	0	0	0	1
0	0	0	0	1
0	0	0	0	1

f) Krankesjön

Sites (columns) × *Species* (rows)

1	0	0	0	0	0
1	0	0	0	0	0
1	0	0	0	0	0
1	0	0	0	0	0
1	1	0	0	0	0
1	1	1	0	0	0
1	1	0	0	0	1
1	1	1	1	0	0
1	1	1	0	1	0
0	1	0	0	0	0
0	1	0	0	0	0
0	1	0	0	0	0
1	1	1	1	1	0
1	1	1	1	1	0
0	1	1	0	0	0
1	1	1	1	1	1
1	0	0	1	1	1
0	0	1	0	0	0
0	0	1	0	0	0
0	1	0	0	1	0
0	1	1	0	1	1
0	1	0	1	0	1
0	1	1	1	1	1
0	1	1	1	1	1
0	1	1	1	1	1
0	1	1	1	1	1
0	0	1	1	0	1
0	0	1	0	1	1
0	0	0	1	0	0
0	0	0	1	0	0
0	0	0	1	0	0
0	0	0	1	1	1
0	0	0	0	1	0
0	0	0	0	1	0
0	0	0	0	1	1
0	0	0	0	1	1

g) Krageholmssjön

Sites (columns) × *Species* (rows)

1	0	0	0	0	0
1	0	0	0	0	0
1	0	0	0	0	0
1	0	0	0	0	0
1	0	0	0	0	0
1	0	0	0	0	0
1	1	1	0	0	0
1	0	1	1	0	0
1	0	1	0	1	0
1	1	1	0	1	1
1	1	1	0	1	1
1	0	1	1	1	1
1	1	1	1	1	1
1	1	1	1	1	1
1	1	1	1	1	1
1	1	1	1	1	1
1	1	1	1	1	1
1	1	1	1	1	1
0	0	1	0	0	0
0	0	1	0	0	0
0	1	1	1	1	0
0	0	1	1	0	0
0	1	1	1	1	1
0	0	1	1	1	0
0	0	1	1	1	0
0	0	1	0	1	1
0	0	0	0	1	0
0	0	0	0	1	1
0	0	0	0	0	1
0	0	0	0	0	1

h) Hökesjön

Sites (columns) × *Species* (rows)

1	0	0	0	0	0	0
1	0	0	0	0	0	0
1	0	0	0	0	0	0
1	0	0	0	0	0	0
1	0	0	0	0	0	0
1	0	0	0	0	0	0
1	0	0	0	0	0	0
1	0	0	0	0	0	0
1	1	0	0	0	0	0
1	1	0	0	0	0	0
1	1	0	0	0	0	0
1	1	1	0	0	0	0
1	1	1	0	0	0	0
1	0	0	0	0	0	1
0	1	0	0	0	0	0
1	1	0	0	1	0	0
1	1	1	0	0	0	1
1	1	1	1	0	0	0
1	1	0	1	0	0	1
1	0	0	1	0	0	1
1	1	1	0	0	1	0
1	0	0	1	0	1	0
1	1	1	0	1	1	1
1	1	1	1	1	1	1
1	1	1	1	1	1	1
1	1	1	1	1	1	1
1	1	1	1	1	1	1
1	1	1	1	1	1	1
1	1	1	1	1	1	1
1	1	0	1	1	1	1
1	1	0	1	1	1	1
0	1	1	1	1	1	1
0	0	0	1	0	0	0
0	0	0	1	0	0	0
0	0	1	0	0	1	1
0	0	0	0	1	0	0
0	0	0	0	1	0	0
0	0	0	0	0	1	0
0	0	0	0	0	1	0
0	0	0	0	0	1	0

S1C Figure. Presence/ absence matrices of Swedish lakes on lake scale. Matrices ordinated using reciprocal averaging.

a) Stechlinsee

Sites (columns) × *Species* (rows)

1	0	0	0	0	0	0
1	0	0	0	0	0	0
1	0	0	0	0	0	0
1	0	0	0	0	0	0
1	0	0	0	0	0	0
1	0	0	0	0	0	0
1	0	0	0	0	0	0
1	1	0	0	0	0	0
1	0	0	0	1	0	0
1	1	1	1	0	0	0
1	1	0	1	1	0	0
0	1	0	0	0	0	0
0	1	0	0	0	0	0
1	1	1	1	1	1	0
1	1	1	1	1	1	0
0	1	0	0	1	0	0
1	1	1	0	1	1	1
1	1	1	1	1	1	1
1	1	1	1	1	1	1
1	1	1	1	1	1	1
1	1	1	1	1	1	1
1	1	1	1	1	1	1
1	1	0	1	1	1	1
0	0	1	0	1	0	0
0	1	0	1	1	1	0
0	0	0	0	1	0	0
0	0	0	0	1	0	0
0	0	0	0	1	0	0
0	0	0	0	1	0	0
0	0	0	1	1	1	0
0	0	0	1	1	1	0
0	0	0	0	0	1	0
0	0	0	0	0	1	1
0	0	0	0	0	0	1
0	0	0	0	0	0	1
0	0	0	0	0	0	1

b) Nehmitzsee

Sites (columns) × *Species* (rows)

1	0	0	0	0	0	0
1	0	0	0	0	0	0
1	1	0	0	0	0	0
1	1	1	0	0	0	0
1	1	1	0	0	0	0
1	1	0	1	0	0	0
1	1	0	1	0	0	0
1	0	0	0	1	0	0
0	1	0	0	0	0	0
1	1	1	1	1	0	0
0	1	1	0	0	0	0
0	1	0	1	0	0	0
0	1	1	1	0	0	0
1	1	0	1	1	1	0
1	1	1	1	1	1	0
1	1	1	1	1	1	0
0	0	1	0	0	0	0
0	0	1	0	0	0	0
0	1	0	1	1	0	0
0	1	0	1	1	0	0
1	1	1	1	1	1	1
1	1	1	1	1	1	1
0	1	1	1	1	1	0
1	1	0	0	1	1	1
0	1	0	0	0	1	0
1	0	1	1	1	1	1
0	1	1	1	0	0	1
0	0	1	1	1	1	0
0	0	1	1	1	1	0
0	0	1	0	0	1	0
0	0	0	1	1	1	0
0	1	0	1	1	1	1
0	1	0	0	1	0	1
0	0	1	1	1	1	1
0	1	0	0	0	0	1
0	0	0	0	0	1	0
0	0	0	0	0	1	0
0	0	0	0	1	0	1
0	0	0	0	0	0	1

c) Haussee

Sites (columns) × *Species* (rows)

1	0	0	0	0	0	0	0
1	0	0	0	0	0	0	0
1	0	0	0	0	0	0	0
1	0	0	0	0	0	0	0
1	1	0	0	0	0	0	0
1	0	0	0	1	0	0	0
1	1	1	0	0	0	0	0
1	0	1	1	1	0	0	0
1	1	1	1	0	1	0	0
1	1	0	0	1	1	1	0
1	1	1	0	1	1	1	0
0	1	0	1	0	0	0	0
1	0	1	1	1	1	1	0
0	1	0	0	0	1	0	0
1	1	1	1	1	1	1	1
1	1	1	1	1	1	1	1
1	1	1	1	1	1	1	1
1	1	1	1	1	1	1	1
0	1	1	1	1	1	0	0
0	0	1	0	1	0	0	0
0	0	0	1	0	0	0	0
0	0	0	0	1	0	0	0
0	0	0	1	1	1	0	0
0	0	1	0	0	0	1	0
0	0	0	0	1	1	0	0
0	0	0	0	0	1	0	0
0	0	0	0	0	1	0	0
0	0	1	0	1	0	1	1
0	0	0	0	0	0	1	0
0	0	0	0	0	0	0	1

d) Tiefer See

Sites (columns) × *Species* (rows)

1	0	0	0	0
1	0	0	0	0
1	0	0	0	0
1	0	0	0	0
1	0	0	0	0
1	0	0	0	0
1	0	0	0	0
1	0	0	0	0
1	0	0	0	0
1	0	0	0	0
1	0	0	0	0
1	0	0	0	0
1	0	0	0	0
1	0	0	0	0
1	0	0	0	0
1	1	0	0	0
1	1	0	0	0
1	1	0	0	0
1	0	1	0	0
1	0	1	0	0
1	0	0	1	0
1	1	0	1	0
1	1	0	0	1
1	0	1	1	0
1	0	1	0	1
1	1	1	1	0
1	1	1	1	0
1	1	1	1	0
1	1	1	1	1
1	1	1	1	1
0	1	0	0	0
0	1	0	0	0
0	1	0	0	0
0	1	0	0	0
0	1	0	0	0
0	1	1	0	0
0	1	1	1	0
0	1	1	1	0
0	1	1	1	0
0	1	1	1	0
0	1	1	0	1
0	0	1	0	0
0	0	1	0	0
0	0	1	1	0
0	0	1	1	0
0	0	1	0	1
0	0	1	1	1
0	0	0	1	0
0	0	0	1	0
0	0	0	1	0
0	0	0	1	0
0	0	0	1	0
0	0	0	0	1
0	0	0	0	1

e) Schöhsee

Sites (columns) × *Species* (rows)

1	0	0	0	0	0	0	0
1	0	0	0	0	0	0	0
1	0	0	0	0	1	0	0
1	1	0	1	0	0	0	0
1	0	1	1	0	0	0	0
1	1	1	0	0	1	0	0
1	1	1	1	0	0	1	0
1	1	1	1	1	1	0	0
0	1	0	0	0	0	0	0
1	1	0	0	1	1	1	0
0	0	1	0	0	0	0	0
0	0	1	0	0	0	0	0
0	0	1	0	0	0	0	0
1	1	1	1	1	1	1	1
1	1	1	1	1	1	1	1
1	0	0	0	1	0	1	1
0	0	0	1	1	0	0	0
0	1	1	1	1	1	1	0
0	1	0	0	0	0	1	0
0	1	1	1	1	1	1	1
0	1	1	1	1	1	1	1
0	1	1	1	0	1	1	1
0	0	0	1	0	0	1	0
0	1	1	0	0	0	1	1
0	0	1	0	0	1	1	1
0	0	0	1	1	0	1	1
0	0	0	1	0	1	1	1
0	0	0	0	0	0	1	0
0	0	0	0	0	1	0	1
0	0	0	0	0	1	0	1
0	0	0	0	0	0	0	1

f) Postsee

Sites (columns) × *Species* (rows)

1	0	0	0	0	0	0
1	0	0	0	0	0	0
1	0	0	0	0	0	0
1	1	0	0	0	0	0
1	0	1	0	1	0	0
1	1	0	1	0	1	0
0	1	0	0	0	0	0
0	1	0	0	0	0	0
0	1	0	1	0	0	0
1	1	1	1	1	0	1
1	1	1	1	1	1	1
1	1	1	1	1	1	1
1	1	1	1	1	1	1
1	1	1	1	1	1	1
1	1	1	1	1	1	1
1	1	1	1	1	1	1
1	1	0	1	1	1	1
0	1	0	1	1	0	1
0	0	0	0	0	1	0
0	0	0	0	1	0	1
0	0	0	0	0	1	1
0	0	0	0	0	0	1

g) Fauler See

Sites (columns) × *Species* (rows)

1	0	0	0
1	0	0	0
1	0	0	0
1	1	0	0
1	1	0	0
1	1	0	0
1	1	0	0
0	1	0	0
0	1	0	0
1	0	1	0
1	1	1	1
1	1	1	1
1	1	1	1
1	1	1	1
1	1	1	1
1	1	1	1
0	1	1	0
0	1	0	1
0	1	0	1
0	1	0	1
0	1	0	1
0	0	1	0
0	0	1	0
0	0	1	0
0	0	1	0
0	0	1	1
0	0	1	1
0	0	1	1
0	0	1	1
0	0	1	1
0	0	0	1
0	0	0	1
0	0	0	1
0	0	0	1
0	0	0	1

h) Löptiner See

Sites (columns) × *Species* (rows)

1	0	0	0	0
1	0	0	0	0
1	1	0	0	0
1	1	1	0	0
0	1	0	0	0
1	1	1	0	1
1	1	1	1	1
1	1	1	1	1
1	1	1	1	1
1	1	1	1	1
0	1	0	0	1
0	1	1	0	1
0	0	1	0	0
0	0	1	1	1
0	0	0	0	1

S1D Figure. Presence/ absence matrices of German lakes on lake scale. Matrices ordinated using reciprocal averaging.

a) Sweden

Sites (columns) / *Species* (rows)

Fiolen	Skärsjön	Hökesjön	Fjärasjö	Älgsjön	Havgardssjön	Krankesjön	Krageholmssjön
1	0	0	0	0	0	0	0
1	0	0	0	0	0	0	0
1	0	0	0	0	0	0	0
1	0	0	0	0	0	0	0
1	0	0	0	0	0	0	0
1	0	0	0	0	0	0	0
1	0	0	0	0	0	0	0
1	0	0	0	0	0	0	0
1	0	0	0	0	0	0	0
1	0	0	0	0	0	0	0
1	0	0	0	0	0	0	0
1	0	0	0	0	0	0	0
1	0	0	0	0	0	0	0
1	0	0	0	0	0	0	0
1	0	0	0	0	0	0	0
1	0	0	0	0	0	0	0
1	0	0	0	0	0	0	0
1	1	0	0	0	0	0	0
1	1	0	0	0	0	0	0
1	1	0	0	0	0	0	0
1	1	0	0	0	0	0	0
1	1	0	0	0	0	0	0
1	1	0	0	0	0	0	0
1	1	0	0	0	0	0	0
1	1	0	0	0	0	0	0
1	1	0	0	0	0	0	0
1	1	0	0	0	0	0	0
1	1	0	0	0	0	0	0
1	0	1	0	0	0	0	0
1	0	1	0	0	0	0	0
1	0	1	0	0	0	0	0
1	1	1	0	0	0	0	0
1	1	1	0	0	0	0	0
1	0	0	1	0	0	0	0
1	1	0	1	0	0	0	0
1	1	0	1	0	0	0	0
1	1	0	1	0	0	0	0
1	1	0	1	0	0	0	0
0	1	0	0	0	0	0	0
0	1	0	0	0	0	0	0
0	1	0	0	0	0	0	0
0	1	0	0	0	0	0	0
0	1	0	0	0	0	0	0
0	1	0	0	0	0	0	0
0	1	0	0	0	0	0	0
0	1	0	0	0	0	0	0
0	1	0	0	0	0	0	0
0	1	0	0	0	0	0	0
1	1	0	0	1	0	0	0
1	1	0	0	1	0	0	0
1	1	1	1	0	0	0	0
1	0	1	1	0	0	0	0
1	1	0	1	1	0	0	0
1	0	0	0	0	1	0	0
1	0	0	0	0	1	0	0
0	1	1	0	0	0	0	0
1	1	0	1	0	0	1	0
0	1	0	1	0	0	0	0
0	1	0	1	0	0	0	0
1	1	1	1	1	0	0	0
1	1	1	1	1	0	0	0
1	1	1	1	1	0	0	0
1	1	1	1	1	0	0	0
1	1	0	0	1	1	0	0
1	1	0	0	1	1	0	0
1	0	1	1	0	0	1	0
1	1	0	0	0	1	1	0
1	1	1	1	1	0	1	0
1	1	0	1	0	1	1	0
0	1	0	1	1	0	0	0
0	0	1	0	0	0	0	0
0	0	1	0	0	0	0	0
0	0	1	0	0	0	0	0
1	1	1	1	1	1	1	0
1	1	1	1	1	1	1	0
0	0	1	1	0	0	0	0
0	0	1	1	0	0	0	0
0	0	1	1	0	0	0	0
0	1	1	1	0	1	0	0
1	1	1	1	1	1	0	1
1	1	0	1	1	1	0	1
0	1	0	0	0	1	0	0
0	1	0	1	0	1	0	0
0	1	0	0	0	0	1	0
0	0	0	1	0	0	0	0
0	0	0	1	0	0	0	0
0	0	0	1	0	0	0	0
0	0	0	1	0	0	0	0
1	1	1	1	0	1	1	1
1	1	1	0	0	1	1	1
1	1	0	0	0	1	1	1
1	1	1	1	1	1	1	1
1	1	1	1	1	1	1	1
1	1	1	1	1	1	1	1
1	1	1	1	1	1	1	1
1	1	1	1	1	1	1	1
1	1	1	1	1	1	1	1
1	1	1	1	1	1	1	1
1	1	1	0	1	1	1	1
1	1	0	1	1	1	1	1
1	1	0	0	1	1	1	1
1	1	0	0	1	1	1	1
0	0	1	1	1	0	0	0
1	0	0	0	1	1	0	1
0	1	0	0	1	0	1	0
1	0	0	0	1	0	1	1
0	1	1	1	1	1	1	0
0	1	0	1	0	1	0	1
0	0	0	1	1	0	0	0
1	0	0	0	1	1	1	1
0	1	0	1	1	1	0	1
0	1	0	0	0	1	0	1
0	1	0	0	0	0	1	1
0	0	1	0	0	0	1	0
0	0	1	0	0	0	1	0
0	0	1	1	1	0	0	1
0	0	0	1	0	0	1	0
0	1	0	0	0	1	1	1
0	0	1	1	1	1	0	1
0	0	0	1	0	0	0	1
0	0	0	0	1	0	0	0
0	0	0	0	1	0	0	0
0	0	0	0	1	0	0	0
0	0	0	0	1	0	0	0
0	0	0	0	1	1	0	0
0	0	0	0	1	0	1	0
0	0	0	0	1	0	1	0
0	0	0	0	0	1	0	0
0	0	0	0	0	1	0	0
0	0	0	0	0	1	0	0
0	0	0	0	0	1	0	0
0	0	0	0	0	1	1	0
0	0	0	0	0	1	1	0
0	0	0	0	0	0	1	0
0	0	0	0	0	0	0	1
0	0	0	0	0	0	0	1
0	0	0	0	0	0	0	1

b) Germany

Sites (columns) / *Species* (rows)

Tiefer See	Nehmitzsee	Fauler See	Haussee	Stechlinsee	Postsee	Schöhsee	Löptiner See
1	0	0	0	0	0	0	0
1	0	0	0	0	0	0	0
1	0	0	0	0	0	0	0
1	0	0	0	0	0	0	0
1	0	0	0	0	0	0	0
1	0	0	0	0	0	0	0
1	0	0	0	0	0	0	0
1	0	0	0	0	0	0	0
1	0	0	0	0	0	0	0
1	0	0	0	0	0	0	0
1	0	0	0	0	0	0	0
1	0	0	0	0	0	0	0
1	0	0	0	0	0	0	0
1	0	0	0	0	0	0	0
1	0	0	0	0	0	0	0
1	1	0	0	0	0	0	0
1	1	0	0	0	0	0	0
1	1	0	0	0	0	0	0
1	1	0	0	0	0	0	0
1	0	1	0	0	0	0	0
1	0	1	0	0	0	0	0
1	1	1	0	0	0	0	0
1	1	1	0	0	0	0	0
0	1	0	0	0	0	0	0
0	1	0	0	0	0	0	0
0	1	0	0	0	0	0	0
0	1	0	0	0	0	0	0
0	1	0	0	0	0	0	0
0	1	0	0	0	0	0	0
1	0	0	1	0	0	0	0
1	0	0	1	0	0	0	0
1	1	1	1	0	0	0	0
1	0	0	0	1	0	0	0
1	1	1	0	1	0	0	0
1	1	1	0	1	0	0	0
1	1	1	1	1	0	0	0
1	1	1	1	1	0	0	0
0	0	1	0	0	0	0	0
0	0	1	0	0	0	0	0
0	0	1	0	0	0	0	0
0	0	1	0	0	0	0	0
0	0	1	0	0	0	0	0
0	1	1	1	0	0	0	0
1	1	1	0	1	0	1	0
1	1	1	1	1	1	0	0
1	1	1	1	1	0	1	0
1	1	1	1	1	0	0	1
1	1	0	1	1	0	1	0
1	1	0	1	1	0	1	0
1	0	1	0	1	0	1	0
0	0	1	0	1	0	0	0
0	0	1	0	1	0	0	0
1	0	0	1	0	0	1	0
1	1	1	1	1	1	1	0
1	1	1	1	1	1	1	0
1	1	1	1	1	1	1	0
0	1	1	0	1	0	1	0
1	1	0	1	1	1	1	0
0	0	0	1	0	0	0	0
0	0	0	1	0	0	0	0
0	0	0	1	0	0	0	0
1	1	1	1	1	1	1	1
1	1	1	1	1	1	1	1
1	1	1	1	1	1	1	1
1	1	1	1	1	1	1	1
1	1	1	1	1	1	1	1
1	1	1	1	1	1	1	1
1	1	1	0	1	1	1	1
0	0	0	0	1	0	0	0
0	0	0	0	1	0	0	0
0	0	0	0	1	0	0	0
0	0	0	0	1	0	0	0
0	0	0	0	1	0	0	0
1	1	0	1	1	1	1	1
1	0	0	1	1	1	1	0
0	0	1	1	0	0	0	1
1	0	1	0	1	1	1	1
0	1	0	1	1	1	1	0
1	0	0	0	0	1	1	1
0	0	0	1	0	1	0	0
0	0	0	0	1	1	1	1
0	0	0	0	0	1	0	0
0	0	0	0	0	1	0	0
0	0	0	0	0	1	1	0
0	0	0	0	0	0	1	0
0	0	0	0	0	0	1	0
0	0	0	0	0	0	1	0
0	0	0	0	0	0	1	0
0	0	0	0	0	0	1	0
0	0	0	0	0	0	1	1
0	0	0	0	0	0	0	1

c) Sweden & Germany

Sites (columns) / *Species* (rows)

Fiolen	Skärsjön	Fjärasjö	Hökesjön	Älgsjön	Havgardssjön	Krankesjön	Fauler See	Krageholmssjön	Nehmitzsee	Tiefer See	Löptiner See	Stechlinsee	Postsee	Schöhsee	Haussee
1	0	0	0	0	0	0	0	0	0	0	0	0	0	0	0
1	0	0	0	0	0	0	0	0	0	0	0	0	0	0	0
1	0	0	0	0	0	0	0	0	0	0	0	0	0	0	0
1	0	0	0	0	0	0	0	0	0	0	0	0	0	0	0
1	0	0	0	0	0	0	0	0	0	0	0	0	0	0	0
1	0	0	0	0	0	0	0	0	0	0	0	0	0	0	0
1	0	0	0	0	0	0	0	0	0	0	0	0	0	0	0
1	0	0	0	0	0	0	0	0	0	0	0	0	0	0	0
1	0	0	0	0	0	0	0	0	0	0	0	0	0	0	0
1	0	0	0	0	0	0	0	0	0	0	0	0	0	0	0
1	0	0	0	0	0	0	0	0	0	0	0	0	0	0	0
1	0	0	0	0	0	0	0	0	0	0	0	0	0	0	0
1	0	0	0	0	0	0	0	0	0	0	0	0	0	0	0
1	0	0	0	0	0	0	0	0	0	0	0	0	0	0	0
1	0	0	0	0	0	0	0	0	0	0	0	0	0	0	0
1	1	0	0	0	0	0	0	0	0	0	0	0	0	0	0
1	1	0	0	0	0	0	0	0	0	0	0	0	0	0	0
1	1	0	0	0	0	0	0	0	0	0	0	0	0	0	0
1	1	0	0	0	0	0	0	0	0	0	0	0	0	0	0
1	1	0	0	0	0	0	0	0	0	0	0	0	0	0	0
1	1	0	0	0	0	0	0	0	0	0	0	0	0	0	0
1	1	0	0	0	0	0	0	0	0	0	0	0	0	0	0
1	1	0	0	0	0	0	0	0	0	0	0	0	0	0	0
1	1	0	0	0	0	0	0	0	0	0	0	0	0	0	0
0	1	0	0	0	0	0	0	0	0	0	0	0	0	0	0
0	1	0	0	0	0	0	0	0	0	0	0	0	0	0	0
0	1	0	0	0	0	0	0	0	0	0	0	0	0	0	0
0	1	0	0	0	0	0	0	0	0	0	0	0	0	0	0
0	1	0	0	0	0	0	0	0	0	0	0	0	0	0	0
0	1	0	0	0	0	0	0	0	0	0	0	0	0	0	0
0	1	0	0	0	0	0	0	0	0	0	0	0	0	0	0
0	1	0	0	0	0	0	0	0	0	0	0	0	0	0	0
0	1	0	0	0	0	0	0	0	0	0	0	0	0	0	0
0	1	0	0	0	0	0	0	0	0	0	0	0	0	0	0
1	1	1	0	0	0	0	0	0	0	0	0	0	0	0	0
1	1	1	0	0	0	0	0	0	0	0	0	0	0	0	0
1	1	0	1	0	0	0	0	0	0	0	0	0	0	0	0
1	1	0	1	0	0	0	0	0	0	0	0	0	0	0	0
1	0	0	1	0	0	0	0	0	0	0	0	0	0	0	0
1	0	0	1	0	0	0	0	0	0	0	0	0	0	0	0
1	1	0	0	1	0	0	0	0	0	0	0	0	0	0	0
0	1	1	0	0	0	0	0	0	0	0	0	0	0	0	0
0	1	1	0	0	0	0	0	0	0	0	0	0	0	0	0
1	0	1	1	0	0	0	0	0	0	0	0	0	0	0	0
0	1	0	1	0	0	0	0	0	0	0	0	0	0	0	0
1	1	1	0	0	0	1	0	0	0	0	0	0	0	0	0
1	1	1	0	0	0	0	1	0	0	0	0	0	0	0	0
1	1	0	0	0	0	0	0	0	1	0	0	0	0	0	0
1	1	0	0	0	0	0	0	0	0	1	0	0	0	0	0
1	0	0	0	0	1	0	0	0	0	0	0	0	0	0	0
1	0	0	0	0	1	0	0	0	0	0	0	0	0	0	0
1	1	0	0	1	1	0	0	0	0	0	0	0	0	0	0
1	1	1	0	0	0	0	0	0	0	1	0	0	0	0	0
0	1	1	0	1	0	0	0	0	0	0	0	0	0	0	0
1	1	1	0	1	0	0	1	0	0	0	0	0	0	0	0
1	1	1	1	0	0	0	0	0	0	1	0	0	0	0	0
1	1	1	1	1	0	0	1	0	0	0	0	0	0	0	0
1	1	1	0	0	1	1	0	0	0	0	0	0	0	0	0
0	0	1	0	0	0	0	0	0	0	0	0	0	0	0	0
0	0	1	0	0	0	0	0	0	0	0	0	0	0	0	0
0	0	1	0	0	0	0	0	0	0	0	0	0	0	0	0
0	1	1	1	0	1	0	0	0	0	0	0	0	0	0	0
1	1	1	1	1	0	0	0	0	0	0	0	1	0	0	0
1	0	0	0	0	0	0	0	0	1	0	0	0	0	0	0
0	0	1	1	0	0	0	0	0	0	0	0	0	0	0	0
0	0	1	1	0	0	0	0	0	0	0	0	0	0	0	0
1	0	1	0	0	0	0	0	0	0	1	0	0	0	0	0
0	1	0	0	0	0	1	0	0	0	0	0	0	0	0	0
1	0	0	1	0	0	0	0	0	1	0	0	0	0	0	0
1	1	1	1	1	0	0	1	0	0	1	0	0	0	0	0
0	0	0	1	0	0	0	0	0	0	0	0	0	0	0	0
0	0	0	1	0	0	0	0	0	0	0	0	0	0	0	0
0	0	1	1	1	0	0	0	0	0	0	0	0	0	0	0
0	1	0	0	1	0	1	0	0	0	0	0	0	0	0	0
0	1	1	0	0	1	0	0	1	0	0	0	0	0	0	0
1	0	0	0	1	1	0	0	1	0	0	0	0	0	0	0
0	1	1	0	1	1	0	0	1	0	0	0	0	0	0	0
1	1	1	1	1	1	1	0	1	0	0	0	0	0	1	0
1	1	0	0	0	1	1	1	0	0	1	0	0	0	0	0
1	1	0	0	1	0	0	1	0	1	1	0	0	0	0	0
0	1	0	0	0	0	1	0	1	0	0	0	0	0	0	0
1	0	1	1	0	0	1	0	0	1	1	0	0	0	0	0
1	1	1	1	1	0	0	1	0	1	1	0	1	0	0	0
1	1	1	0	1	1	0	1	1	1	1	0	0	0	0	0
1	0	0	0	0	0	0	1	0	0	0	0	1	0	0	0
0	0	0	0	1	0	0	0	0	0	0	0	0	0	0	0
0	0	0	0	1	0	0	0	0	0	0	0	0	0	0	0
0	0	0	0	1	0	0	0	0	0	0	0	0	0	0	0
0	0	0	0	1	0	0	0	0	0	0	0	0	0	0	0
0	0	1	0	0	0	0	0	1	0	0	0	0	0	0	0
0	0	0	1	0	0	1	0	0	0	0	0	0	0	0	0
1	1	1	0	1	1	1	1	1	1	0	0	0	0	0	1
1	1	1	1	1	0	1	1	0	1	1	0	1	0	1	0
0	0	0	0	1	1	0	0	0	0	0	0	0	0	0	0
0	0	1	0	0	0	0	0	0	0	1	0	0	0	0	0
0	1	1	1	1	1	1	1	0	1	1	0	1	0	0	0
0	0	1	1	0	0	0	0	0	1	1	0	0	0	0	0
1	1	1	1	1	1	1	0	0	1	0	0	1	1	1	1
1	1	1	1	1	1	1	1	0	1	1	0	1	0	1	1
1	1	1	1	1	1	0	1	1	1	1	0	1	1	0	1
0	0	0	0	1	0	1	0	0	0	0	0	0	0	0	0
0	0	0	0	1	0	1	0	0	0	0	0	0	0	0	0
0	0	1	1	1	1	0	0	1	1	1	0	0	0	0	0
1	1	0	0	1	1	0	0	0	0	0	1	1	1	1	0
1	1	1	1	1	1	1	0	1	0	1	0	1	1	1	1
1	1	0	0	1	1	1	0	1	0	1	1	0	1	1	0
1	1	1	1	1	1	1	1	1	1	1	0	1	1	1	1
0	0	0	0	0	1	0	0	0	0	0	0	0	0	0	0
0	0	0	0	0	1	0	0	0	0	0	0	0	0	0	0
0	0	0	0	0	1	0	0	0	0	0	0	0	0	0	0
0	0	0	0	0	1	0	0	0	0	0	0	0	0	0	0
0	1	1	0	0	1	0	1	0	0	1	0	1	0	1	0
1	1	0	1	1	1	1	1	1	1	1	1	1	1	1	0
1	1	1	1	1	1	1	1	1	1	1	1	1	1	1	1
1	1	1	1	1	1	1	1	1	1	1	1	1	1	1	1
1	1	1	1	1	1	1	1	1	1	1	1	1	1	1	1
1	1	1	1	1	1	1	1	1	1	1	1	1	1	1	1
1	1	0	0	1	1	1	1	1	0	1	1	1	1	1	0
1	1	1	1	0	1	1	1	1	1	1	1	1	1	1	1
0	1	0	0	0	1	1	0	1	0	1	0	0	0	0	1
0	1	0	0	0	1	0	1	0	0	0	1	0	0	0	1
0	0	1	1	1	0	0	1	1	1	1	0	0	0	0	1
1	1	0	1	0	1	1	1	1	1	1	1	1	1	1	1
0	0	0	0	0	0	1	0	0	0	0	0	0	0	0	0
1	1	0	0	0	1	1	0	1	1	1	0	1	1	1	1
0	0	0	0	0	0	0	1	0	0	0	0	0	0	0	0
0	0	0	0	0	0	0	1	0	0	0	0	0	0	0	0
1	0	0	0	1	0	1	1	1	1	1	1	1	0	0	1
0	1	0	0	0	1	0	1	1	1	1	0	1	0	0	1
0	0	0	0	0	0	0	0	1	0	0	0	0	0	0	0
0	0	0	0	0	0	0	0	1	0	0	0	0	0	0	0
0	0	1	0	0	0	1	1	0	1	0	0	1	0	1	0
0	0	1	0	1	0	0	0	0	0	1	0	0	0	1	1
1	0	0	0	1	1	1	0	1	1	1	1	1	1	1	1
0	0	0	1	0	0	1	0	0	0	0	0	0	1	1	0
0	0	0	0	0	0	0	0	1	0	1	0	0	0	0	0
0	0	0	0	0	0	0	0	0	1	0	0	0	0	0	0
0	0	0	0	0	0	0	0	0	1	0	0	0	0	0	0
0	0	0	0	0	0	0	0	0	1	0	0	0	0	0	0
0	0	0	0	0	0	0	0	0	1	0	0	0	0	0	0
0	0	0	0	0	0	0	1	0	0	0	0	1	0	0	0
0	0	0	0	0	0	0	0	0	1	1	0	0	0	0	0
0	0	0	1	0	0	0	1	0	1	1	0	1	1	1	1
0	0	0	0	0	1	1	1	0	1	1	0	1	1	1	1
0	0	0	0	0	1	1	0	0	1	1	0	1	0	1	1
0	0	0	0	0	0	0	0	0	0	1	0	0	0	0	0
0	0	0	0	0	0	0	0	0	0	1	0	0	0	0	0
0	0	0	0	0	0	0	0	0	0	1	0	0	0	0	0
0	0	0	0	0	0	0	0	0	0	1	0	0	0	0	0
0	0	0	0	0	0	0	0	0	0	1	0	0	0	0	0
0	0	0	0	0	0	0	0	0	0	1	0	0	0	0	0
0	0	0	0	0	0	0	0	0	0	1	0	0	0	0	0
0	0	0	0	0	0	0	0	0	0	1	0	0	0	0	0
0	0	0	0	0	0	0	0	0	0	1	0	0	0	0	0
0	0	0	0	0	0	0	1	0	1	1	0	1	0	0	1
0	0	0	0	0	0	0	0	0	0	1	0	1	0	0	0
0	0	0	0	0	0	0	0	0	0	0	1	0	0	0	0
0	0	0	0	0	0	0	0	0	1	1	0	1	0	1	1
0	0	0	0	0	0	0	0	0	0	0	0	1	0	0	0
0	0	0	0	0	0	0	0	0	0	0	0	1	0	0	0
0	0	0	0	0	0	0	0	0	0	0	0	1	0	0	0
0	0	0	0	0	0	0	0	0	0	0	0	1	0	0	0
0	0	0	0	0	0	0	0	0	0	1	0	0	0	0	1
0	0	0	0	0	0	0	0	0	0	0	0	0	1	0	0
0	0	0	0	0	0	0	0	0	0	0	0	0	1	0	0
0	0	0	0	0	0	0	0	0	0	0	1	0	0	1	0
0	0	0	0	0	0	0	0	0	0	0	0	0	0	1	0
0	0	0	0	0	0	0	0	0	0	0	0	0	0	1	0
0	0	0	0	0	0	0	0	0	0	0	0	0	0	1	0
0	0	0	0	0	0	0	0	0	0	0	0	0	0	1	0
0	0	0	0	0	0	0	0	0	0	0	0	0	1	0	1
0	0	0	0	0	0	0	0	0	0	0	0	0	0	0	1
0	0	0	0	0	0	0	0	0	0	0	0	0	0	0	1
0	0	0	0	0	0	0	0	0	0	0	0	0	0	0	1

S1E Figure. Presence/ absence matrices of Swedish (a) and German lakes (b) on regional scale and on supra-regional scale (c). Matrices ordinated using reciprocal averaging.
