# Supplementary material for: Varying Patterns on Varying Scales: A Metacommunity Analysis of Nematodes in European Lakes
Source: PLoS One. 2016 Mar 23;11(3):e0151866. doi: 10.1371/journal.pone.0151866 (PMC4805234; doi:10.1371/journal.pone.0151866)
Supplement: S1 Table — (DOCX) [file pone.0151866.s002.docx]

**Supplementary data**

S1 Table. Nematode species found in the 16 sampled lakes in Sweden and Germany. Identification of species and of feeding types was made by Walter Traunspurger.

| **Species** | **Feeding type** |
| --- | --- |
| *Achromadora* cf. *ruricola* (De Man 1880) | e |
| *Achromadora longicauda* (Schneider 1937) | e |
| *Achromadora micoletzkyi* (Stefanski 1915) | e |
| *Achromadora tenax* (De Man 1876) | e |
| *Achromadora terricola* (De Man 1880) | e |
| *Alaimus parvus* (Thorne 1939) | d |
| *Alaimus primitivus* (De Man 1880) | d |
| *Amphidelus elegans* (De Man 1921) | d |
| *Anatonchus dolichurus* (Ditlevsen 1911) | r |
| *Anonchus mirabilis* (Hofmänner in Hofmänner & Menzel 1914) | d |
| *Aphanolaimus aquaticus* (Daday 1894) | d |
| *Aphanolaimus viviparus* (Plotnikoff 1899) | d |
| *Aphelenchoides* sp. 1 | s-p |
| *Aphelenchoides* sp. 2 | s-p |
| *Aporcelaimellus obtusicaudatus* (Bastian 1865) | s-o |
| *Aporcelaimellus* sp. | s-o |
| *Bastiania longicaudata* (De Man 1880) | d |
| *Bastiania* sp. 1 | d |
| *Bastiania* sp. 2 | d |
| *Brevitobrilus stefanskii* (Micoletzky 1925) | o |
| *Calolaimus ditlevseni* (Micoletzky 1922) | s-o |
| *Cephalobus persegnis* (Bastian 1865) | d |
| *Chromadorina bercziki* (Andrássy 1962) | e |
| *Chromadorina bioculata* (Schultze in Carus 1857) | e |
| *Chromadorina viridis* (Linstow 1876) | e |
| *Chromadorita leuckarti* (De Man 1876) | e |
| *Chronogaster tenuis* (Loof & Jairajpuri 1965) | d |
| *Chronogaster typica* (De Man 1921) | d |
| *Chrysonema attenuatum* (De Man 1880) | s-o |
| *Chrysonema limigenum* (Siddiqi 1969) | s-o |
| *Clarkus papillatus* (Bastian 1865) | r |
| *Crocodorylaimus flavomaculatus* (Linstow 1876) | s-o |
| *Cryptonchus tristis* (Ditlevsen 1911) | d |
| *Cylindrolaimus communis* (De Man 1880) | d |
| *Diplogaster rivalis* (Leydig 1854) | o |
| *Diplogasteritus* sp. | o |
| *Ditylenchus acutatus* (Brzeski 1991) | s-p |
| Dorylaimidae 1 (De Man 1876) | s-o |
| Dorylaimidae 2 (De Man 1876) | s-o |
| *Dorylaimoides* cf. *ariasae* (Loof 1990) | s-o |
| *Dorylaimus* sp. | s-o |
| *Dorylaimus stagnalis* (Dujardin 1845) | s-o |
| *Doryllium* cf. *zeelandicum* (De Man 1876) | s-o |
| *Epidorylaimus* cf. *agilis* (De Man 1880) | s-o |
| *Epidorylaimus consobrinus* (De Man 1918) | s-o |
| *Epitobrilus* cf. *husmanni* (Altherr 1958) | o |
| *Ethmolaimus* cf. *parapratensis* (Alekseev, Naumova & Dymina 1979) | e |
| *Ethmolaimus pratensis* (De Man 1880) | e |
| *Ethmolaimus* sp. | e |
| *Eudorylaimus carteri* (Bastian 1865) | s-o |
| *Eudorylaimus* cf. *similis* (De Man 1876) | s-o |
| *Eudorylaimus* sp. | s-o |
| *Eumonhystera* cf. *barbata* (Andrássy 1981) | d |
| *Eumonhystera* cf. *simplex* (De Man 1880) | d |
| *Eumonhystera dispar* (Bastian 1865) | d |
| *Eumonhystera filiformis* (Bastian 1865) | d |
| *Eumonhystera longicaudatula* 1 (Gerlach & Riemann 1973) | d |
| *Eumonhystera longicaudatula* 2 (Gerlach & Riemann 1973) | d |
| *Eumonhystera pseudobulbosa* (Daday 1896) | d |
| *Eumonhystera* sp. 1 | d |
| *Eumonhystera* sp. 2 | d |
| *Eumonhystera* sp. 3 | d |
| *Eumonhystera vulgaris* (De Man 1880) | d |
| *Euteratocephalus palustris* (De Man 1880) | d |
| *Eutobrilus altherri* (Andrássy 1959) | o |
| *Eutobrilus* cf. *graciliformis* (Altherr in Altherr & Delamare Deboutteville 1972) | o |
| *Eutobrilus* cf. *grandipapillatus* (Brakenhoff 1914) | o |
| *Eutobrilus medius* (Schneider 1916) | o |
| *Eutylenchus excretorius* (Ebsary & Eveleigh 1981) | s-p |
| *Filenchus vulgaris* (Brzeski 1963) | s-p |
| *Helicotylenchus* cf. *vulgaris* (Yuen 1964) | s-p |
| *Helicotylenchus* sp. | s-p |
| *Hemicycliophora thienemanni* (Schneider 1925) | s-p |
| *Hemicycliophora typica* (De Man 1880) | s-p |
| *Heterocephalobus elongatus* (De Man 1880) | d |
| *Hirschmaniella gracilis* (De Man 1880) | s-p |
| *Iotonchus* sp. | r |
| *Ironus ignavus* (Bastian 1865) | r |
| *Ironus longicaudatus* (De Man 1884) | r |
| *Ironus tenuicaudatus* (De Man 1876) | r |
| *Ironus terranovus* (Ebsary 1985) | r |
| *Ischiodorylaimus* sp. | s-o |
| *Laimydorus flevensis* (Loof 1996) | s-o |
| *Laimydorus parabastiani* (Paetzold 1958) | s-o |
| *Laimydorus* sp. | s-o |
| *Lelenchus* sp. | s-p |
| *Lenonchium* sp. | s-o |
| *Malenchus* cf. *neosulcus* (Geraert & Raski 1986) | s-p |
| Mermithidae | o |
| *Mesodorylaimus* cf. *bastiani* (Bütschli 1873) | s-o |
| *Mesodorylaimus conurus* (Thorne 1939) | s-o |
| *Mesodorylaimus* sp. 1 | s-o |
| *Mesodorylaimus* sp. 2 | s-o |
| *Mesodorylaimus* sp. 3 | s-o |
| *Mesodorylaimus subtiliformis* (Andrássy 1959) | s-o |
| *Metateratocephalus crassidens* (De Man 1880) | d |
| *Monhystera* cf. *lemani* (Juget 1969) | d |
| *Monhystera paludicola* (De Man 1881) | d |
| *Monhystera* sp. 1 | d |
| *Monhystera* sp. 2 | d |
| *Monhystera stagnalis* (Bastian 1865) | d |
| *Monhystrella paramacrura* (Meyl 1953) | d |
| *Mononchus aquaticus* (Coetzee 1968) | r |
| *Mononchus maduei* (Schneider 1925) | r |
| *Mononchus niddensis* (Skwarra 1921) | r |
| *Mononchus truncatus* (Bastian 1865) | r |
| *Mononchus tunbridgenesis* (Bastian 1865) | r |
| *Mylonchulus* cf. *brevicaudatus* (Cobb 1917) | r |
| *Mylonchulus* cf. *lacustris* (Cobb in Cobb 1915) | r |
| *Neoactinolaimus duplicidentatu* (Andrássy 1968) | s-o |
| *Neoactinolaimus* sp. | s-o |
| *Neotobrilus* cf. *longus* (Leidy 1852) | o |
| *Panagrolaimus* sp. | d |
| *Paractinolaimus macrolaimus* (De Man 1880) | s-o |
| *Paramphidelus* sp. 1 | d |
| *Paramphidelus uniformis* (Thorne 1939) | d |
| *Paraphanolaimus anisitsi* (Daday 1905) | d |
| *Plectus aquatilis* (Andrássy 1985) | d |
| *Plectus cf. acuminatus* (Bastian 1865) | d |
| *Plectus* cf. *cirratus* (Bastian 1865) | d |
| *Plectus* cf. *longicaudata* (Bütschli 1873) | d |
| *Plectus* cf. *opisthocirculatus* (Andrássy 1952) | d |
| *Plectus geophilus* (De Man 1880) | d |
| *Plectus palustris* (De Man 1880) | d |
| *Plectus rhizophilus* (De Man 1880) | d |
| *Plectus* sp. | d |
| *Plectus tenuis* (Bastian 1865) | d |
| Pratylenchidae 1 | s-p |
| *Pratylenchus* sp. | s-p |
| *Prismatolaimus dolichurus* (De Man 1880) | e |
| *Prismatolaimus intermedius* (Bütschli 1873) | e |
| *Prodesmodora arctica* (Mulvey 1969) | e |
| *Prodesmodora circulata* (Micoletzky 1913) | e |
| *Prodesmodora terricola* (Altherr 1952) | e |
| *Prodorylaimus acris* (Thorne 1939) | s-o |
| *Prodorylaimus brzeskii* (Winiszewska-Slipinska 1987) | s-o |
| *Prodorylaimus rotundiceps* (Loof 1985) | s-o |
| *Prodorylaimus* sp. | s-o |
| *Punctodora ratzeburgensis* (Linstow 1876) | e |
| Rhabditidae 1 | d |
| Rhabditidae 2 | d |
| *Rhabditis* sp. | d |
| *Rhabdolaimus aquaticus* (De Man 1880) | d |
| *Rhabdolaimus* sp. 2 | d |
| *Rhabdolaimus terrestris* (De Man 1880) | d |
| *Rhabdolaimus terrestris/ aquaticus* (De Man 1880) | d |
| *Semitobrilus* cf. *longicaudatus* (Hofmänner 1913) | o |
| *Semitobrilus pellucidus* (Bastian 1865) | o |
| *Teratocephalus terrestris* (Bütschli 1873) | d |
| *Theristus agilis* (De Man 1880) | d |
| *Theristus vesentinae* (Andrássy 1962) | d |
| *Thonus* sp. | s-o |
| *Thornia* sp. 1 | s-o |
| *Thornia* sp. 2 | s-o |
| *Tobrilus gracilis* (Bastian 1865) | o |
| *Tobrilus* sp. 1 | o |
| *Tobrilus* sp. 2 | o |
| *Tripyla affinis* (De Man 1880) | o |
| *Tripyla cornuta* (Skwarra 1921) | o |
| *Tripyla glomerans* (Bastian 1865) | o |
| *Tripyla setifera* (Bütschli 1873) | o |
| *Trischistoma monohystera* (De Man 1880) | o |
| Tylenchidae | s-p |
| *Tylencholaimus* cf. *mirabilis* (Bütschli 1873) | s-o |
| *Tylencholaimus* cf. *proximus* (Thorne 1939) | s-o |
| *Tylenchorhynchus* sp. | s-o |
| *Tylenchus* sp. 2 | s-p |
| *Tylenchus* sp. 3 | s-p |
| *Tylenchus* sp. 4 | s-p |
| *Tylenchus* sp. 5 | s-p |
| *Udonchus tenuicaudatus* (Cobb 1913) | d |
| Unknown species 1 | - |
| Unknown species 2 | - |
| Unknown species 3 | - |

e, epistrate feeder; d, deposit feeder; r, predator /chewer; s-p, stylet/fungus/ herbivore; s-o, stylet/ omnivore; o, omnivore
